# Transarterial chemoembolization as a substitute to radiofrequency ablation for treating Barcelona Clinic Liver Cancer stage 0/A hepatocellular carcinoma

**DOI:** 10.18632/oncotarget.25108

**Published:** 2018-04-20

**Authors:** Kentaro Ishikawa, Tetsuhiro Chiba, Yoshihiko Ooka, Eiichiro Suzuki, Sadahisa Ogasawara, Takahiro Maeda, Masayuki Yokoyama, Masanori Inoue, Toru Wakamatsu, Yuko Kusakabe, Tomoko Saito, Akinobu Tawada, Makoto Arai, Tatsuo Kanda, Hitoshi Maruyama, Fumio Imazeki, Naoya Kato

**Affiliations:** ^1^ Department of Gastroenterology, Graduate School of Medicine, Chiba University, Chiba, Japan

**Keywords:** hepatocellular carcinoma, transarterial chemoembolization, radiofrequency ablation, Barcelona Clinic Liver Cancer (BCLC) staging, propensity score matching

## Abstract

**Background and Aim:**

Transarterial chemoembolization (TACE) is the standard procedure for treating Barcelona clinic liver cancer (BCLC) stage B hepatocellular carcinoma (HCC). However, it is often carried out in the treatment of BCLC stage 0/A HCC for various reasons. This study aimed to elucidate the prognosis for BCLC stage 0/A HCC patients treated with TACE or with radiofrequency ablation (RFA).

**Materials and Methods:**

The prognosis of 242 BCLC stage 0/A HCC patients within Milan criteria who underwent initially TACE or RFA were retrospectively analyzed using propensity score matching analysis.

**Results:**

The analyses of baseline patient characteristics revealed that the maximum tumor size and the proportion of BCLC stage A patients were significantly higher in patients treated with TACE than in those treated with RFA (*P*<0.001 and 0.047, respectively). After adjusting these factors using propensity score matching (1:3 matching), patients treated with TACE (n=32) and those treated with RFA (n=96) were further analyzed. The local recurrence rate was significantly higher in the TACE group than in the RFA group (*P*<0.001). However, the overall survival (OS) in HCC patients treated with TACE was comparable to that in HCC patients treated with RFA (1 year, 93.5 vs. 95.8%; 3 years, 75.4 vs. 85.8%; 5 years, 61.8 vs. 70.7%; *P*=0.196). Multivariate analyses followed by univariate analyses revealed that serum bilirubin level (*P*=0.032), serum albumin level (*P*=0.008), HBV-DNA (*P*=0.013), and tumor number (*P*=0.021) were independent predictors of OS.

**Conclusion:**

TACE can substitute RFA at least in some patients with BCLC 0/A HCC.

## INTRODUCTION

Hepatocellular carcinoma (HCC) is the sixth most common carcinoma worldwide and is the third leading cause of cancer-related mortality [1]. Every year, more than 700,000 new cases of HCC are reported, and its incidence rate has been gradually increasing [2]. Approximately 90% of HCC develop in cirrhosis caused by hepatitis viral infection, alcohol consumption, and metabolic syndrome [3]. Staging systems for HCC, such as the Okuda staging system and TNM classification, have been utilized to comprehend the clinical manifestation and predict the prognosis of HCC patients [4, 5]. The Barcelona Clinic Liver Cancer (BCLC) staging system classifies HCC into 0, A, B, C, and D based on not only the extent of tumor but also Child-Pugh score, and the Eastern Cooperative Oncology Group (ECOG) performance status [6, 7]. The best feature of this classification system is that treatment modalities are strictly defined for each stage. For example, HCC patients in Child-Pugh A with a single lesion or three lesions within 30 mm diameter are categorized in BCLC stage A (early stage). Of these, patients with a single lesion less than 20 mm in diameter are classified as BCLC stage 0. For patients with BCLC stage 0/A HCC, local treatment techniques, such as hepatic resection or ablation therapy, are recommended [8–10]. Conversely, BCLC stage B HCC comprises multiple tumors without extrahepatic metastasis or macrovascular invasion. Transarterial chemoembolization (TACE) is considered as the standard treatment option for these tumors [8–10]. Although TACE is occasionally performed for BCLC stage 0/A HCC patients for various reasons in clinical practice, its significance remains unclear.

In this study, we retrospectively compared the overall survival (OS) between BCLC stage 0/A HCC patients within the Milan criteria treated initially with radiofrequency ablation (RFA) or with TACE and evaluated prognostic factors for these patients.

## PATIENTS AND METHODS

### Patients

The medical records of patients were retrieved from those who underwent TACE or RFA as an initial treatment for HCC at our institution from January 2009 to December 2014. A total of 414 patients who were histologically or radiologically diagnosed with HCC based on the diagnostic criteria of the American Association for the Study of Liver Diseases were identified [8]. Of these, patients in BCLC stage 0/A within the Milan criteria but not in BCLC stage B, C or D were enrolled. This study was approved by the Research Ethics Committees of Graduate School of Medicine, Chiba University (Chiba, Japan; approval number 2,762).

### Treatment

TACE was conducted as previously described [11]. Briefly, superior mesenteric arteriography and common hepatic arteriography were initially performed to assess vascular abnormalities, tumor burden, tumor vascularity, and portal vein patency. Thereafter, an emulsion of anticancer drugs such as cisplatin (Nippon Kayaku, Tokyo, Japan), epirubicin (Nippon Kayaku) and miriplatin (Dainippon Sumitomo Pharma Co., Ltd., Osaka, Japan) in iodized oil (Lipiodol; Laboratoire Guerbet, Aulnay-Sous-Bois, France) was infused into the selected feeding artery, which was followed by embolization with 1-mm-diameter absorbable gelatin sponge particles (Gelfoam; Upjohn, Kalamazoo, MI) until arterial flow stasis was achieved.

RFA was performed as previously described [12]. Briefly, procedures were performed under real-time ultrasound guidance (Power Vision 8000, Aplio XV, Aplio XG, or Aplio 500; Toshiba, Tokyo, Japan) and a 17-gauge cooled-tip electrode (Cool-Tip; RF Ablation System, Covidien, Boulder, Colombia, CO). Under conscious sedation with local anesthesia, an electrode was inserted and radiofrequency waves were delivered for 6-15 min for each lesion. If necessary, intrapleural or intraperitoneal fluid infusion was performed before electrode insertion to more precisely recognize tumors and maintain distance between the tumor and the intestinal tract. The effectiveness was evaluated by performing dynamic computed tomography (CT) or magnetic resonance imaging (MRI) one day after treatment. To assess the completeness of ablation, the images taken before and after ablation were compared.

### Assessment of antitumor effect of TACE

In this study, the therapeutic responses of TACE were evaluated on the basis of the dynamic CT or dynamic MRI findings three months after TACE according to the assessment of the modified Response Evaluation Criteria in Solid Tumors (mRECIST) [13]. According to the mRECIST criteria, complete response (CR) was defined as the disappearance of contrast enhancement during the arterial phase in all target lesions. Partial response (PR) and progressive disease (PD) were defined as at least a 30% decrease and as at least a 20% increase in the sum of the diameters of viable enhanced tumors, respectively. Stable disease (SD) was defined as any case that qualified for neither PR nor PD.

### Statistical analysis

Statistical differences between RFA and TACE groups were compared using Student's *t*-test, and the chi-square test for categorical data. To minimize the bias for patient selection, propensity score matching (1:3) analyses were used to compare the two groups. OS, recurrence-free survival (RFS), and progression-free survival (PFS) were calculated using the Kaplan-Meier method and compared using the log-rank test. Univariate and multivariate Cox regression analyses were conducted to determine the prognostic relevance of clinical variables. Variables associated with OS in univariate analysis with *P*<0.20 were retained in the multivariate analysis. *P*<0.05 was considered statistically significant.

## RESULTS

### Patient characteristics

**Figure 1:**
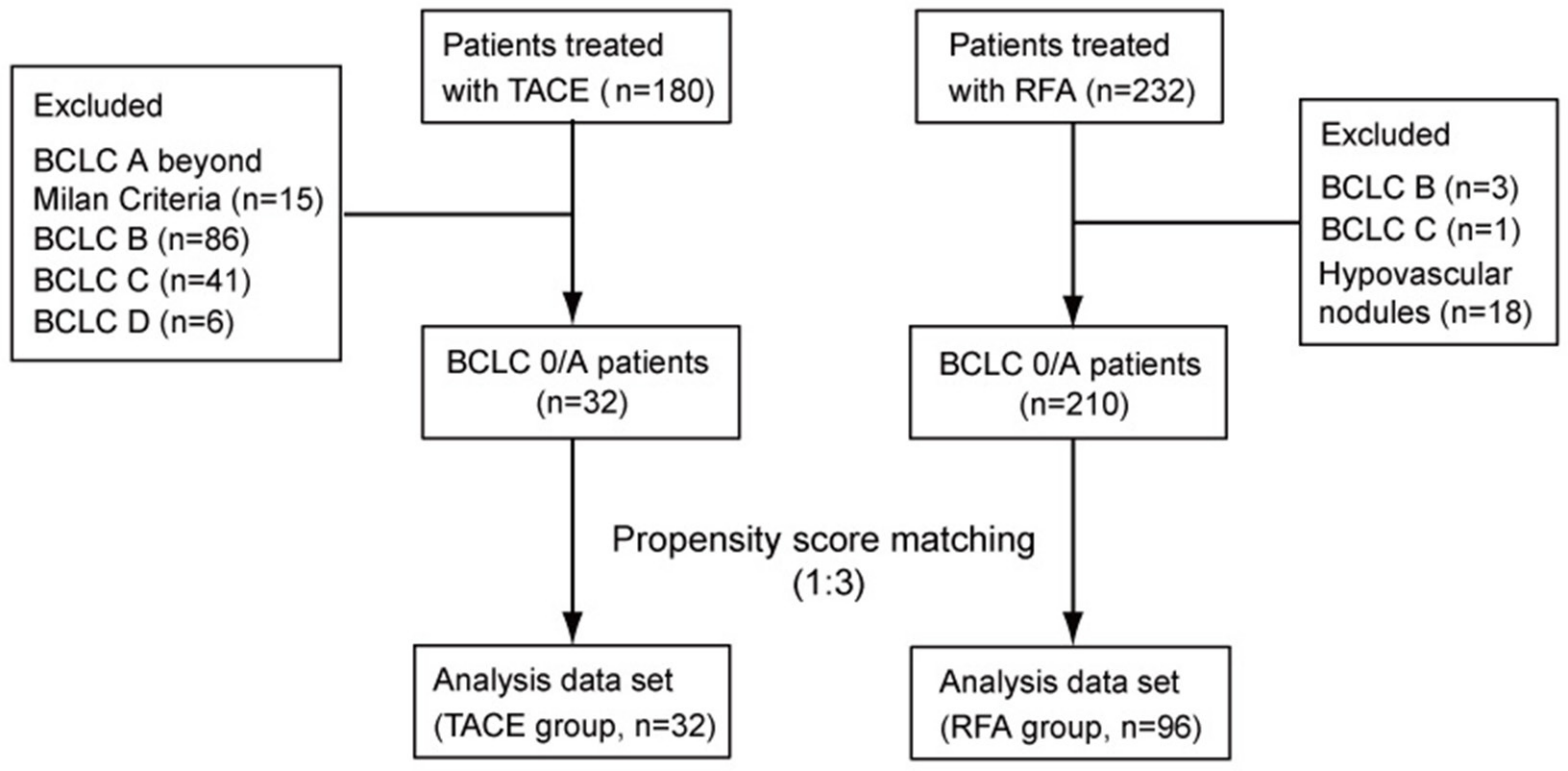
Patients enrolled in this study

Of 180 patients treated with TACE as a first treatment for HCC, 32 were categorized into BCLC stage 0/A stage within Milan Criteria. Similarly, of 232 patients initially treated with RFA, 228 were categorized into BCLC stage 0/A. We excluded 18 patients with hypovascular nodules who were treated with RFA. Then, 32 patients treated with TACE (TACE group) and 210 patients treated with RFA (RFA group) were further analyzed (Figure 1). The reasons for avoiding RFA in 32 patients in the TACE group were: not eligible for hepatectomy because of unfavorable estimated remnant liver function (7 patients), location of the tumor near the vascular channel or another organ (7), patient intention (6), inadequate extraction during ultrasonography (2), ascites (2), hemodialysis (1), advanced obesity (1), decreased respiratory function (1), and other (5).

Subsequently, clinical variables were compared between the TACE and RFA groups (Table 1). There were no significant differences between the two groups in terms of age, sex, aspartate aminotransferase (AST) level, total bilirubin level, albumin level, platelet count, hepatitis B virus (HBV)-DNA positivity, hepatitis C virus (HCV)-RNA positivity, Child–Pugh grade, indocyanine green retention rate at 15 minutes (ICG-R15), tumor number, and alpha-fetoprotein (AFP) level. However, patients in the TACE group had significantly larger tumors than those in the RFA groups (*P*<0.001). The proportion of BCLC stage 0 HCC patients was considerably lower in the TACE group than in the RFA group (*P*=0.044). Although there was no significant difference, compared with patients in the RFA groups, those in the TACE group demonstrated a trend of high serum level of total bilirubin (*P*=0.113).

**Table 1:** Baseline patient characteristics

Variable	TACE group (n=32)	RFA group (n=210)	*P*-value
Age (median, years)	71 (48-88)	71 (37-85)	0.545
Sex (male/female)	19/13	131/79	0.896
AST (median, IU/L)	45 (12-207)	51 (9-174)	0.161
Total bilirubin (median, mg/dL)	1.1 (0.4-2.4)	1.0 (0.4-3.1)	0.150
Albumin (median, g/dL)	3.8 (2.7-4.9)	3.7 (2.5-4.8)	0.860
Platelet count (median, x10^4^/uL)	10.1 (2.8-34.3)	9.5 (3.1-29.6)	0.891
HBV DNA (negative/positive)	29/3	194/16	0.731
HCV RNA (negative/positive)	16/16	88/122	0.503
Child-Pugh grade (A/B)	22/10	162/48	0.416
ICG-R15 (median, %)	25.5 (6.8-57.1)	23.0 (3.6-74.8)	0.578
Tumor number (solitary/multiple)	24/8	150/60	0.836
Maximal tumor size (median, mm)	26 (8-45)	18 (5-38)	<0.001^*^
AFP (median, ng/mL)	15.0 (2.5-4,306)	12.7 (1.6-12,916)	0.152
BCLC stage (0/A)	4/28	66/144	0.047^*^

^*^Statistically significant. AST, aspartate aminotransferase; HBV, hepatitis B virus; HCV, hepatitis C virus; ICG-R15, retention rate of indocyanine green 15 min after administration; AFP, alpha-fetoprotein; BCLC, Barcelona Clinic Liver Cancer.

### Propensity score matching model

To reduce selection bias, 96 of 210 patients in the RFA group were matched with 32 patients in the TACE group using propensity score matching (Supplementary Table 1). After propensity score matching, there were no significant differences between the two groups regarding age, sex, AST level, total bilirubin level, albumin level, platelet count, HBV-DNA positivity, HCV-RNA positivity, Child-Pugh grade, ICG-R15, maximal tumor size, tumor number, AFP level, and BCLC stage 0/A proportion.

### OS of patients treated with TACE or RFA

**Figure 2:**
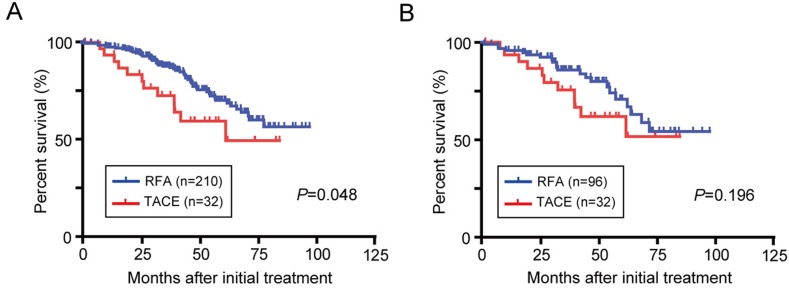
OS in BCLC stage 0/A HCC patients initially treated with TACE or RFA Kaplan-Meier survival curves in all study patients **(A)** and after propensity score matching **(B)**.

The median follow-up period in the entire cohort and propensity score matched cohort were 43.0 months and 40.0 months, respectively (Figure 2). In the entire cohort, the median OS was significantly longer in the RFA group than in the TACE group (*P*=0.048). However, there was no significant difference in the median OS between the TACE and RFA groups in propensity score matched cohort (*P*=0.196). The 1-, 3-, and 5-year cumulative OS rates in the propensity score model were 93.5%, 75.4%, and 61.8% in the TACE group, and 95.8%, 85.8%, and 70.7% in the RFA group. In the TACE group, 7/32 patients died from cancer progression (4 patients), liver failure (2), and other non-liver-related causes (1). Additionally, 54/210 patients in the RFA group perished because of cancer progression (18 patients), liver failure (16), other non-liver-related causes (12), unknown causes (5) and gastroesophageal bleeding (3).

### Recurrence in patients treated with TACE or RFA

**Figure 3:**
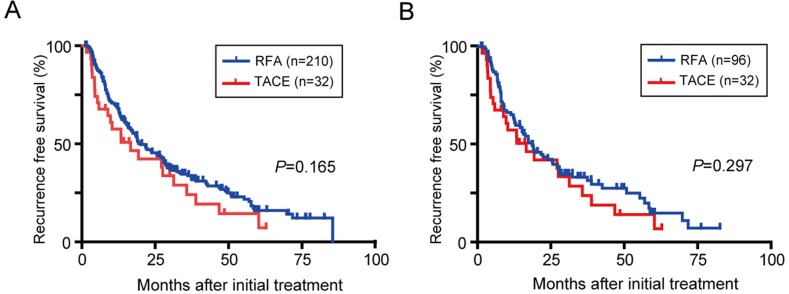
RFS in BCLC stage 0/A HCC patients Kaplan-Meier survival curves in all study patients **(A)** and after propensity score matching **(B)**.

In the RFA group, 152/210 and 69/96 patients with HCC exhibited recurrence in the entire and the propensity score matched cohort, respectively. Similarly, 24/32 patients in the TACE group also showed recurrence. The median RFS in the TACE group was comparable to that of the RFA group in both in the entire cohort and in the propensity score matched cohort (Figure 3; *P*=0.165 and *P*=0.297, respectively). The 1-, 3-, and 5-year cumulative RFS rates in the propensity score cohort were 57.6%, 24.2%, and 14.5% in the TACE group, and 65.5%, 33.6%, and 15.3% in the RFA group. Of the 24 recurrent patients in the TACE group, the subsequent treatments included: TACE (14 patients), RFA (7), PEIT (1), systemic chemotherapy (1), and best supportive care (1). In the 69 patients in the RFA group, the subsequent treatments were: RFA (43 patients), TACE (19), PEIT (2), and best supportive care (5).

The numbers of the additional treatment per year between the patients treated with RFA and TACE in the propensity matched cohort was 0.36±0.06 and 0.53±0.13, respectively (*P*=0.171). Likewise, the rate per two years was 0.83±0.10 and 0.97±0.24, respectively (*P*=0.550).

### Local recurrence and salvage therapy after TACE or RFA

**Figure 4:**
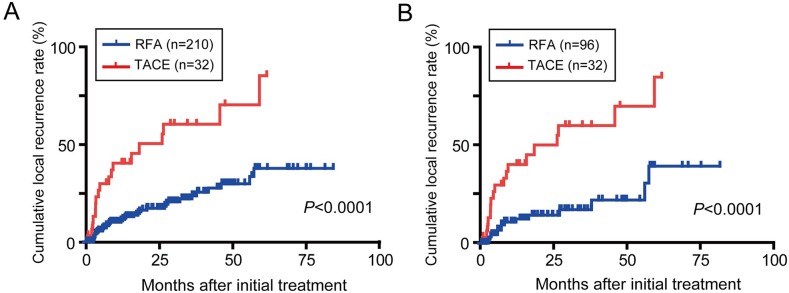
Cumulative local recurrence rates in BCLC stage 0/A HCC patients Kaplan-Meier survival curves in all study patients **(A)** and after propensity score matching **(B)**.

Local recurrence was observed in 38/210 and 16/96 patients treated with RFA in the entire cohort and the propensity matched cohort, respectively (Figure 4). In the TACE group, 18/32 patients exhibited local recurrence. The cumulative local recurrence rates in the patients treated with TACE was significantly higher than that observed in the patients treated with RFA (1 year, 40.3 vs. 10.1%; 3 years, 60.2 vs. 22.2%; 5 years, 70.2 vs. 37.6%; *P*<0.001).

Twelve patients in both groups were treated with additional curative RFA as a salvage therapy. The post-progression survival (PPS) in the patients of the TACE group was similar to that of patients of the RFA group (1 year, 100.0 vs. 100.0%; 3 years, 76.2 vs. 79.5%; 5 years, 50.8 vs. 59.7%; *P*=0.726). In addition, PFS in 24 patients treated with additional RFA against local recurrence lesions was higher than that in patients without such treatment (*P*=0.092).

### Relationship between the effect of TACE and OS

**Figure 5:**
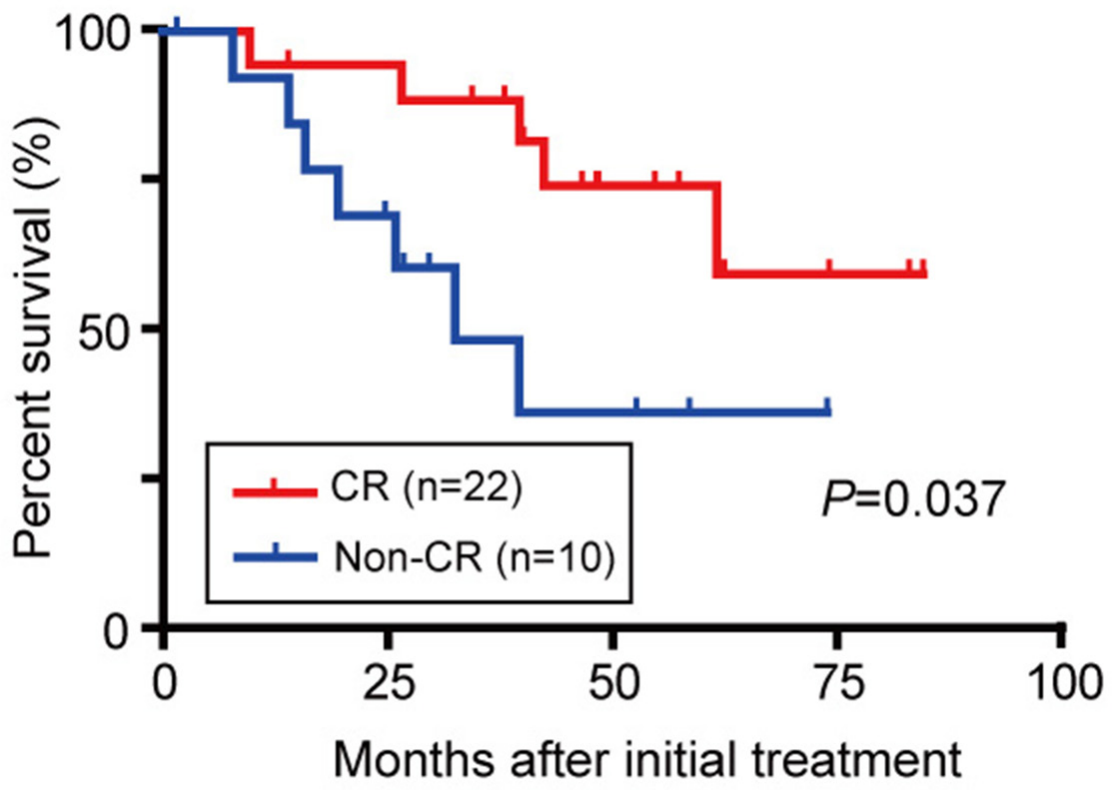
OS in BCLC stage 0/A HCC patients initially treated with TACE considering the treatment response

Treatment effects were also evaluated three months after TACE by follow up dynamic CT or dynamic MRI according to the mRECIST criteria. The number of patients presenting with CR, PR, SD, and PD was 22 (68.8%), 5 (15.6%), 3 (9.4%), and 2 (6.3%), respectively. In contrast, 205 out of 210 patients (97.6%) in RFA group exhibited CR. Then, the relationship between the effect of TACE and OS was examined. The Kaplan-Meier survival analyses revealed that the patients with CR demonstrated a significantly longer OS than those with non-CR (Figure 5; *P*=0.037). These findings indicate that a favorable treatment effect of TACE is closely associated with the survival advantage. In particular, these patients with CR in TACE group was characterized by tumor location in peripheral segments (II/III and VI/VII) of the liver compared to patients with non-CR.

### Prognostic factors for survival

Univariate and multivariate Cox regression analyses were conducted to examine the prognostic relevance of clinical variables in propensity score matched cohort (Table 2). Variables with *P*<0.20 in the univariate analysis (total bilirubin level, serum albumin level, AST level, HBV-DNA, HCV-RNA, Child-Pugh grade, tumor number, and BCLC stage) were subjected to the multivariate analysis. Multivariate Cox's regression analyses revealed that the total bilirubin level, serum albumin level, HBV-DNA, and tumor number served as statistically significant predictors of OS. The analyses in the entire cohort revealed that the total bilirubin level, serum albumin levels, and HBV-DNA, but not tumor number, were determined as prognostic factors of survival (Supplementary Table 2).

**Table 2:** Prognostic factors for the overall survival in the propensity score matched cohort

	Univariate analysis		Multivariate analysis	
	Hazard ratio (95% CI)	*P*-value	Hazard ratio (95% CI)	*P*-value
Age (≥70 years)	1.382 (0.685-2.811)	0.364		
Sex (male)	0.833 (0.412-1.760)	0.622		
Total bilirubin (>ULN)	3.661 (1.761-8.329)	<0.001^*^	2.393 (1.078-5.744)	0.032^*^
Albumin (<LLN)	4.807 (2.021-14.168)	<0.001^*^	4.335 (1.447-15.681)	0.008^*^
AST (>ULN)	2.805 (1.101-9.472)	0.029^*^	1.231 (0.381-4.887)	0.741
Platelet count (≤1×10^5^/μL)	1.360 (0.679-2.855)	0.390		
HBV DNA (positive)	0.288 (0.016-1.354)	0.134	0.144 (0.008-0.719)	0.013^*^
HCV RNA (positive)	1.679 (0.820-3.699)	0.160	1.308 (0.602-3.082)	0.508
Child-Pugh grade (grade B)	3.211 (1.617-6.535)	<0.001^*^	0.864 (0.454-2.047)	0.733
Tumor number (multiple)	2.923 (1.451-5.808)	0.003^*^	2.509 (1.155-5.431)	0.021^*^
Maximal tumor size (≥20mm)	0.688 (0.335-1.378)	0.267		
AFP (≥20ng/mL)	1.202 (0.590-2.390)	0.604		
BCLC (stage A)	8.341 (1.794-148.387)	0.003^*^	4.633 (0.832-87.724)	0.086
Therapeutic approach (TACE)	1.643 (0.767-3.320)	0.193	2.073 (0.930-4.397)	0.074

^*^Statistically significant. ULN, upper limit of normal; LLN, lower limit of normal; AST, aspartate aminotransferase; HBV, hepatitis B virus; HCV, hepatitis C virus; AFP, alpha-fetoprotein; BCLC, Barcelona Clinic Liver Cancer, TACE, transarterial chemoembolization.

## DISCUSSION

RFA is a minimally invasive and an effective local treatment method for curative intent in HCC patients [14]. According to the guidelines of the Liver Cancer Study Group of Japan, RFA is applied for patients with three or fewer tumors of ≤3 cm in diameter [15]. However, complications such as bleeding, dissemination, abscesses, hepatic infarcts, digestive tract perforation, and portal vein thrombosis have also been reported [16, 17]. Furthermore, it has been reported that the amount of heat through radiofrequency is reduced when a tumor is located near the blood vessel, resulting in inadequate ablation [18]. Thirthy-two patients in the TACE group in this study avoided RFA for vairous reasons, and this prompted us to examine the prognosis of these patients.

BCLC stage 0/A HCC patients included patients with single and multiple HCCs. Jin et al. and Zhu et al. analyzed BCLC stage A HCC patients with tumors measuring ≥5 cm in diameter (exceeding the Milan criteria) and reported that patients who were operated on had a significantly better OS than those who underwent TACE [19, 20]. Hence, it is better to consider resection for a large single HCC rather than loco-regional treatment including TACE and RFA. Therefore, in our study, we investigated BCLC stage 0/A HCC patients who met the Milan criteria. Patients in the TACE group tended to have larger tumor diameters and fewer BCLC stage 0 tumors than those in the RFA group, and it appears essential to correct for these biases. When we used propensity score matching to lower selection bias, we discovered that there was no significant difference in OS between the two groups.

Our results are concordant with reports by other groups. Chen et al. examined BCLC stage 0/A HCC patients undergoing RFA and TACE (103 each) using propensity score matching and found no significant difference in OS [21]. In their study, the mean age of the patients was approximately 50 years. Ninety percent of their patients exhibited hepatitis B-related HCC (B-HCC), and Child Pugh B and HCV-positivity were determined to be factors related to poor prognosis. Hsu et al. examined HCC patients within the Milan criteria and studied patients undergoing RFA and those undergoing TACE (101 each) using propensity score matching and found no significant difference in OS [22]. In this research, inadequate PS (≥1) and vascular invasion were associated with poor prognosis. In a sub-analysis, RFA appeared superior in terms of local recurrence rate and total survival rate when the total tumor volume was ≤11cm^3^. Considering that the local recurrence rate in TACE-treated patients is significantly higher than that observed in RFA-treated patients [23], these findings seem reasonable. Reportedly, tumor response has been considered as one of the factors for prognosis in TACE [24, 25]. In our study, OS was better in CR cases than in non-CR cases, suggesting that it is profoundly involved in long-term prognosis when therapeutic effects are favorable. Although local recurrence was frequently observed in the TACE group compared to the RFA group, OS of patients with salvage therapy after local recurrence was longer than that of all patients in the TACE group. These findings indicate that initial treatment and additional curative treatments such as RFA and surgery, are essential for improving prognosis.

Concordant with our previous report [26], we found in the present study that the number of tumors was detected as one of the prognosticators. Regarding the therapeutic effects of RFA and TACE on a single HCC measuring ≤2 cm in diameter, it has been reported that there was no significant difference in OS, even though the time to progression was longer in the RFA group [27]. Guideline of the Japanese Society of Hepatology recommend local ablation therapy for the BCLC stage 0/A HCC patients based on the findings that the patients within Milan criteria treated with TACE showed higher local recurrence rates than patients treated with RFA. However, RFA against HCCs >30mm in diameter sometimes requires increased ablation sessions and ablation areas, which might cause RFA-induced complications. We previously determined that early-stage HCC patients with RFA-induced complications such as bleeding and intrahepatic bile duct damage contributes exhibit poorer prognosis [12]. Overall, RFA should be considered as a first-line therapy for patients with early stage HCC (≤30mm in diameter) who do not have any specific reason for avoiding RFA.

Liver function in chronic liver disease patients is commonly assessed by the Child–Pugh score. In recent years, however, the new albumin–bilirubin (ALBI) grade, which is used for evaluating liver function without peritoneal effusion and encephalopathy and bases the assessment on albumin and bilirubin levels only, has been proposed [28]. The ALBI grade is a useful prognosis predictor even in cases of liver resection or in cases in which the progressive stage is being treated with sorafenib [29, 30]. Consistent with these reports, we detected albumin and bilirubin as prognosticators not by the Child–Pugh score. These results suggest that even if a case is Child–Pugh A, the prognosis might be poor if serum albumin and/or bilirubin levels are abnormal.

Chronic infection by HBV or HCV is one of the leading causes of the development of HCC in patients worldwide. Reportedly, the prognosis of B-HCC was better than that of hepatitis C-related HCC (C-HCC). This finding is, in large part, attributable to the decline in the viral overload by interferon and nucleos(t)ide analog use [31, 32]. Similarly, HBV-positivity was detected as a better prognostic factor in our study. In other words, this suggests that the prognosis of C-HCC cases is poor. Elimination of the virus was achieved in only a handful of patients with HCV infection receiving conventional interferon (IFN)-based therapy because of side effects and the low sweep efficiency. However, owing to the recent availability of direct acting antivirals (DAAs), HCV eradication has become safe and almost absolute [33]. While improvement in hepatic function and the suppression of hepatocarcinogenesis is expected by HCV eradication, it has been reported that the recurrence rate of viral infection after virus eradication by DAA is significantly high in postoperative patients with HCC [34]. Further comparison and examination of the prognosis of C-HCC patients who underwent virus eradication by DAA or IFN-based therapy are needed in the future.

This study had limitations. First, this was retrospective study with a relatively small cohort, Second, propensity score matching analysis cannot completely remove selection bias. Further analyses in larger number of patients is necessary to clarify this issue.

In conclusion, this study demonstrated that the prognosis of patients treated with TACE is not necessarily inferior than that of patients treated with RFA. Hence, TACE serves as an efficient substitute to RFA at least in some patients who are ineligible for ablation therapy.

## SUPPLEMENTARY MATERIALS TABLES



## Abbreviations

HCC: hepatocellular carcinoma
BCLC: Barcelona Clinic Liver Cancer
ECOG: Eastern Cooperative Oncology Group
TACE: transarterial chemoembolization
OS: overall survival
RFA: radiofrequency ablation
mRECIST: modified Response Evaluation Criteria in Solid Tumors
CT: computed tomography
MRI: magnetic resonance imaging
CR: complete response
PR: partial response
PD: progressive disease
SD: stable disease
RFS: recurrence-free survival
PFS: progression-free survival
AST: aspartate aminotransferase
HBV: hepatitis B virus
HCV: hepatitis C virus
AFP: alpha-fetoprotein
ICG-R15: indocyanine green retention rate at 15 minutes
ALBI: albumin–bilirubin
IFN: interferon
DAA: direct acting antiviral.
