# Supplementary material for: Transarterial chemoembolization as a substitute to radiofrequency ablation for treating Barcelona Clinic Liver Cancer stage 0/A hepatocellular carcinoma
Source: Oncotarget. 2018 Apr 20;9(30):21560–8. doi: 10.18632/oncotarget.25108 (PMC5940395; doi:10.18632/oncotarget.25108)
Supplement: Supplementary file 1 [file oncotarget-09-21560-s001.pdf]

Transarterial chemoembolization as a substitute to radiofrequency ablation for treating Barcelona Clinic Liver Cancer stage 0/A hepatocellular carcinoma

SUPPLEMENTARY MATERIALS

Supplementary Table 1: Patient characteristics in the propensity score matched cohort

Variable	TACE group (n=32)	RFA group (n=96)	*P*-value
Age (median, years)	71 (48-88)	69 (37-83)	0.806
Sex (male/female)	19/13	62/34	0.751
AST (median, IU/L)	45 (12-207)	50 (9-174)	0.122
Total bilirubin (median, mg/dL)	1.1 (0.4-2.4)	1.1 (0.4-3.1)	0.951
Albumin (median, g/dL)	3.8 (2.7-4.9)	3.7 (2.5-4.7)	0.819
Platelet count (median, $\times 10^4$/uL)	10.1 (2.8-34.3)	8.8 (3.2-28.4)	0.887
HBV DNA (negative/positive)	29/3	89/7	0.704
HCV RNA (negative/positive)	16/16	40/56	0.537
Child-Pugh grade (A/B)	22/10	65/31	0.913
ICG-R15 (median, %)	25.5 (6.8-57.1)	24.9 (5.1-74.8)	0.690
Tumor number (solitary/multiple)	24/8	72/24	1.000
Maximal tumor size (median, mm)	26 (8-45)	22 (13-38)	0.324
AFP (median, ng/mL)	15.0 (2.5-4,306)	12.7 (1.8-12,916)	0.596
BCLC stage (0/A)	4/28	20/76	0.433

AST, aspartate aminotransferase; HBV, hepatitis B virus; HCV, hepatitis C virus; ICG-R15, retention rate of indocyanine green 15 min after administration; AFP, alpha-fetoprotein; BCLC, Barcelona Clinic Liver Cancer.

Supplementary Table 2: Prognostic factors for the overall survival in the entire cohort

	Univariate analysis		Multivariate analysis	
	**Hazard ratio (95% CI)**	***P*-value**	**Hazard ratio (95% CI)**	***P*-value**
Age (≥70 years)	1.203 (0.737-1.987)	0.461		
Sex (male)	1.263 (0.755-2.188)	0.381		
Total bilirubin (>ULN)	2.320 (1.422-3.782)	<0.001[*]	2.146 (1.170-3.961)	0.014[*]
Albumin (<LLN)	2.534 (1.486-4.537)	<0.001[*]	1.998 (1.065-3.885)	0.031[*]
AST (>ULN)	1.731 (0.942-3.493)	0.079	0.953 (0.479-2.046)	0.897
Platelet count ($\leq 1\times10^5/\mu L$)	1.469 (0.897-2.453)	0.127	0.784 (0.446-1.401)	0.405
HBV DNA (positive)	0.271 (0.044-0.869)	0.024[*]	0.244 (0.039-0.834)	0.021[*]
HCV RNA (positive)	2.116 (1.229-3.843)	0.006[*]	1.722 (0.944-3.300)	0.077
Child-Pugh grade (grade B)	2.345 (1.417-3.827)	0.001[*]	1.169 (0.625-2.211)	0.627
Tumor number (multiple)	1.621 (0.965-2.662)	0.067	1.507 (0.860-2.612)	0.149
Maximal tumor size (≥20mm)	1.086 (0.666-1.771)	0.741		
AFP (≥20ng/mL)	1.632 (0.997-2.662)	0.051	1.143 (0.678-1.923)	0.614
BCLC (stage A)	2.485 (1.294-5.385)	0.005[*]	1.370 (0.629-3.227)	0.437
Therapeutic approach (TACE)	1.586 (0.786-2.915)	0.186	1.413 (0.673-2.735)	0.344

[*]Statistically significant. ULN, upper limit of normal; LLN, lower limit of normal; AST, aspartate aminotransferase; HBV, hepatitis B virus; HCV, hepatitis C virus; AFP, alpha-fetoprotein; BCLC, Barcelona Clinic Liver Cancer, TACE, transarterial chemoembolization.
